# A Systematic Review of Patient Satisfaction With Removable Partial Dentures (RPDs)

**DOI:** 10.7759/cureus.51793

**Published:** 2024-01-07

**Authors:** Mohammed Awawdeh, Meshari B Alotaibi, Abdualrhman H Alharbi, Sultan A Alnafisah, Turki S Alasiri, Naif Ibrahim Alrashidi

**Affiliations:** 1 College of Dentistry, King Saud bin Abdulaziz University for Health Sciences (KSAU-HS), Riyadh, SAU; 2 King Abdullah International Medical Research Center, Ministry of National Guard Health Affairs, Riyadh, SAU; 3 Dental Services, King Abdulaziz Medical City, Ministry of National Guard Health Affairs, Riyadh, SAU; 4 College of Medicine and Dentistry, Ulster University, Birmingham, GBR; 5 Dental Services King Abdulaziz Medical City, Ministry of National Guard Health Affairs, Riyadh, SAU

**Keywords:** outcomes, satisfaction rate, patient satisfaction, satisfaction, aesthetics, chewing ability, partial edentulous, removable denture, partial denture, removable partial denture

## Abstract

Removable partial dentures (RPDs) offer a broad range of aesthetics and restorative functions for partially edentulous patients. This systematic review examines patients' satisfaction rates and the factors that influence RPD satisfaction.

This systematic review was conducted according to PRISMA (Preferred Reporting Items for Systematic Reviews and Meta-Analyses) guidelines and the Cochrane Handbook for Systematic Reviews. A systematic literature search was done on PubMed, Scopus, and Google Scholar using preset inclusion criteria.

A total of 923 non-duplicate articles were screened, and 35 were included in this review. Among the included studies, RPDs generally exhibited high satisfaction rates, with reported rates ranging between 50% and 81%. Several factors influenced satisfaction. Age played a major role, with older adults expressing higher satisfaction. Gender differences were also noted, especially in appearance satisfaction where women were more satisfied with RPDs than men. Prior experience with RPDs correlated positively with overall satisfaction. The number and location of missing teeth, as well as the type of RPD (metal vs. flexible), significantly influenced satisfaction levels. Flexible dentures were more satisfactory than metal RPDs. Attachments, such as magnetic attachments and implants, increased satisfaction. Patient complaints, encompassing pain, aesthetics, and cleanliness, were identified as common sources of dissatisfaction.

The results underscore the significance of customizing RPD treatment to individual needs, considering factors that influence RPD satisfaction. Recognizing the importance of influential factors such as age, gender RPD experience, etc., for clinicians seeking to optimize patient outcomes in RPD therapy is crucial.

## Introduction and background

A removable partial denture (RPD) is designed for partially edentulous patients in need of teeth replacement for either functional or aesthetic purposes [1]. This option is suitable when a fixed partial denture (bridge) is not feasible due to factors such as insufficient supporting teeth (distal abutments) or financial constraints. There are three types of RPDs: cast metal partial denture, flexible partial denture, and acrylic partial denture (flipper tooth). RPDs are supported by the surrounding tissues and remaining teeth [2]. They are equipped with clasps made of cobalt-chrome, titanium metal, or plastic, which securely attach to the existing teeth, thereby enhancing the stability and retention of the RPD [3-4].

RPDs offer a broad spectrum of restorative functions. These include maintaining or enhancing speech clarity, improving masticatory efficiency, stabilizing dental relationships, and achieving the desired aesthetics [1,5-8]. Despite these benefits, RPDs pose a challenge of dissatisfaction for several patients. The biggest concern is usually the aesthetic aspect, which impacts both appearance and interpersonal communication [9-10]. Patient dissatisfaction with RPDs also stems from concerns about potential local damage to remaining teeth, such as risks of caries, periodontal disease, plaque accumulation, oral candidiasis, and denture stomatitis [2,4,11-14].

Due to the potential dissatisfaction associated with RPDs, one key focus during their design should be to ensure a patient’s satisfaction. In literature, satisfaction with RPDs is reported to be influenced by various factors, including the unique characteristics of each patient, their attitude toward RPD, prior experiences with removable dentures, the level of encouragement received for denture use, and the specific design and fabrication process of the RPD [3,15,16]. This review aims to improve knowledge of the factors influencing satisfaction with RPDs. Its primary objective is to assess the satisfaction rates among patients using RPDs. Additionally, the review investigates the various factors that contribute to satisfaction levels with RPDs.

## Review

Protocol

This systematic review was conducted in accordance with the PRISMA (Preferred Reporting Items for Systematic Reviews and Meta-Analyses) recommendations [17] and the Cochrane Handbook for Systematic Reviews. This study utilizes data collected from published clinical studies. Its design does not require any form of approval from an ethics committee.

Outcomes

The participants included all patients wearing RPDs, and the intervention focused on those who underwent rehabilitation with RPDs. Comparison or lack thereof was not a matter of concern as the review sought to understand the subjective experiences of patients utilizing RPDs. The primary outcome of studies was the patient-reported outcome measure of patient satisfaction evaluated after RPD therapy. A population, intervention, control, and outcome (PICO) were used to formulate a primary outcome question: What is the satisfaction rate among patients wearing RPDs? Patient satisfaction is the sense of well-being that patients feel following prosthetic therapy.

Inclusion and exclusion criteria

The articles included in this study were carefully selected to fulfill the study's purpose and address the following research question: "What is the prevalence of satisfaction in patients wearing RPDs? The inclusion and exclusion criteria were meticulously established and adhered to to achieve this objective. The review included clinical trials and observational studies evaluating patient-reported satisfaction outcomes associated with RPDs. Articles from studies with no available data, prosthetic rehabilitations other than RPDs, clinical report cases, case series, and reviews of all kinds were excluded from the study. Similarly, studies that did not assess RPD in terms of satisfaction or with fewer than 20 participants were excluded from the review. The cap for the number of participants was not considered for randomized controlled trials. Also, there is no time limit, all the articles published up to 2023 were included. No specific language was selected; all articles meeting the inclusion criteria were accepted, irrespective of the original language in which the articles were written.

Search strategy and databases

Three databases (MEDLINE/PubMed, Scopus, and Google Scholar) were electronically searched to identify all the relevant studies for articles published up to 2023 with no date language limitations. A supplemental manual search was performed on reference lists of included articles. The following keywords combined with Boolean operators and Medical Subject Headings (MeSH) were used in PubMed and modified for Scopus and Google Scholar (["removable partial denture" OR "partial denture" OR "removable denture" OR "partial edentulous" OR Retention OR "Chewing ability" OR Aesthetics OR "Buccal clasps") AND (satisfaction OR "patient satisfaction" OR "satisfaction rate" OR "patient experience" OR "quality of life" OR PROMs) AND ("dental patients" OR "denture wearers" OR "partial edentulism" OR edentulism) AND ("prosthodontic outcomes" OR "quality of life" OR "outcomes"]).

Selection procedure and data extraction

Reviewers performed a methodical analysis of all study titles, abstracts, and full text. Any disagreements were resolved by discussion to find a consensus during study selection and data extraction. The selection of studies at the database level was performed in the following steps. First, the retrieved articles were imported into a reference management software program (Zotero; Corporation for Digital Scholarship, Vienna, VA), and duplicates were subsequently removed. The remaining studies were then imported into Rayyan (Rayyan Systems, Inc.) where title and abstract screening was performed. The third step involved reading the full text of the selected studies and applying the inclusion and exclusion criteria. Studies not meeting the inclusion criteria were excluded, and the reasons for exclusion were recorded. Data extraction and synthesis were performed including collecting the following data: author and year of publication, study design, number and age of participants, type of denture, follow-up period, satisfaction variables considered, and the main results.

Results

Search Results

**Figure 1:**
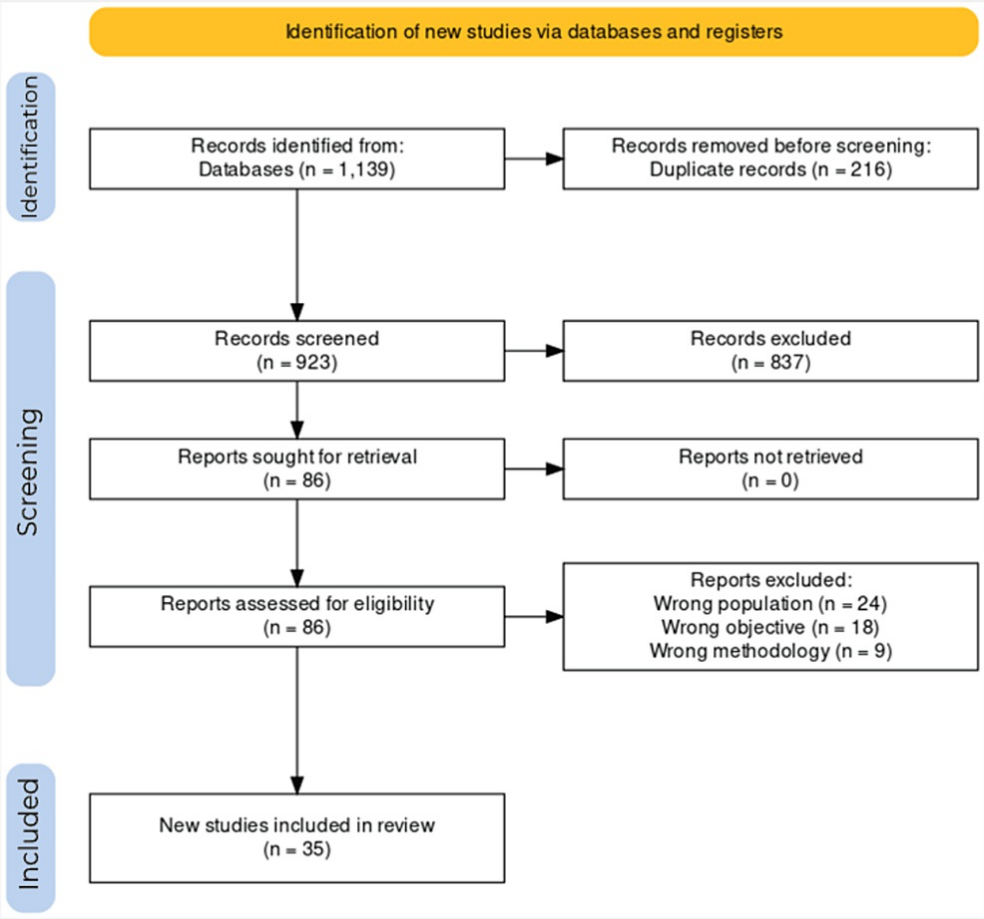
PRISMA flowchart. PRISMA, Preferred Reporting Items for Systematic Reviews and Meta-Analyses

A bibliographic search of PubMed and Scopus databases identified 1,139 relevant articles where 216 articles were duplicates. A manual search was done on Google Scholar, where the first 10 pages were considered. The first stage of screening resulted in excluding 837 articles based on title and/or abstract screening. After title and abstract screening, 86 articles remained and were read in full. Fifty-one articles were eliminated based on the inclusion and exclusion criteria; the reasons for exclusion are included in the PRISMA flowchart (Figure 1). Thirty-five articles were finally included that had a clear assessment of the satisfaction of patients with RPDs.

Results of data extraction

The data extraction results are shown in the study descriptor table (Table 1).

**Table 1:** Study descriptor table. VAS, visual analog scale; PMMA, polymethyl methacrylate; FPD, fixed partial denture; RPD, removable partial denture; OHRQoL, oral health-related quality of life; TR-RPD, thermoplastic resin-removable partial denture; MC-RPD, conventional metal clasp-retained removable partial denture; IARPD, implant-assisted removable partial denture; MI, mini implant; SD, standard deviation; CD, complete denture

Author and year	Study design	Sample size	Age range/mean	Gender distribution	Denture	Comparison	Classification	Outcome measures	Outcomes (primary or secondary)	Findings	Country of origin
Yoshimoto et al. (2021) [18]	Retrospective cross-sectional	132	71.0 ± 9.0	55 men	RPD	-	Kennedy classification II (over 40%)	VAS, Gummy Jelly, and food acceptance status	Masticatory satisfaction	Masticatory satisfaction among RPD wearers was not significantly associated with gender, age, denture wearing jaw, Kennedy classification, and occlusal support. Mean masticatory satisfaction score (VAS) = 75.3 across all participants.	Japan
Aljabri et al. (2017) [19]	Retrospective study	60	51.18 ± 13.06	30 men	RPD (PMMA-based, nylon-based, and chrome cobalt alloys)	-		Satisfaction Questionnaire	Patient satisfaction	35% were very satisfied, 21.7% were satisfied, and 23.3% were nearly satisfied.	Saudi Arabia
Fueki et al. (2018) [20]	Randomized cross-over trial	24	67.3	36.0% men	TR-RPDs, MC-RPDs	-	-	VAS, Likert Scale	Satisfaction, denture-related parameters	TR-RPDs hold an advantage over MC-RPDs in terms of oral appearance and can offer greater satisfaction than MC-RPDs in partially dentate arches with at least an occluding pair in the posterior region. The mean (±SD) overall satisfaction scores were 87.3 (±15.5) and 81.0 (±17.4) for TR-RPDs and MC-RPDs, respectively.	Japan
Shala et al. (2016) [21]	Retrospective study	63	61.4 ± 9.6	36 men	RPDs, RPDs with attachment	-	Kennedy class I (34), IA [11], II [10]	Satisfaction Questionnaire	Patient satisfaction	73.6% of patients were wearing RPD for the first time and were finally satisfied. According to the denture support of RPDs, clasp-retained quadrangular RPDs were 100% effective, followed by triangular dental support at 81% and linear dental support at 47.7%.	Kosovo
Akinyamoju et al. (2017) [22]	Quasi-experimental study	30	33.8 ± 10.01	-	flexible design, acrylic	-	-	VAS, questionnaire	Appearance, ease of cleaning, ability to speak, comfort while eating, and overall satisfaction	Subjects were more satisfied with the flexible RPD than the acrylic resin RPD. 21 (70.0%) participants were more satisfied with the flexible dentures, 6 (20.0%) with acrylic dentures, while 3 (10.0%) were equally satisfied with both types of dentures (*P* = 0.04).	Nigeria
Bilhan et al. (2012) [23]	Retrospective study	99	63.26 ± 9.6	44 men	RPD			VAS	Patient satisfaction with several data about the dentures such as denture age, type of denture, centric relation, and vertical dimension	Loss of retention, ulcerations, and high vertical dimension caused patient dissatisfaction. Additionally, dentures with wrong-centric relations caused the need for the addition of artificial teeth.	Turkey
Negoro et al. (2021) [24]	Prospective study	27	70.8 (7.1/48-83)	11 men	IARPDs (short implants with magnetic attachments)	-	Kennedy Class I [21] and Class II [9]	VAS	Genera satisfaction, patient denture assessment and OHRQoL	The PDA, general patient satisfaction, and OHRQoL were improved by IARPD with a short implant using a magnetic attachment	Japan
Al Jaghsi et al. (2020) [25]	Multi-center randomized clinical trial	76	43-83	29 men	Double-crown-retained, clasp-retained, attachment-retained			Satisfaction Questionnaire	General satisfaction, RPD retention, stability, support, eating, speaking, and aesthetics	Strategic MIs improved the satisfaction of patients with RPDs during the medium-term follow-up period. An earlier improvement in the satisfaction of patients with RPDs was seen after immediate loading of the MIs as compared with delayed loading.	Germany
Koyama et al. (2009) [26]	Retrospective study	67	66.0-9.5	18	RPD	-	-	Satisfaction Questionnaire	Satisfaction outcomes	The continued utilization of RPDs is related to factors such as the patient’s age, location of the edentulous area, number of occluding pairs of teeth, and number of occlusal rests, satisfaction, including pain while using RPDs, the color of the artificial teeth, and tooth shape and setup.	Japan
Frank et al. (1998) [27]	Population-based study		58.6 ± 10.9	49.8% women	RPD (Mandibular)	-	-	Satisfaction Questionnaire	Satisfaction outcomes	A majority of survey respondents treated with a mandibular RPD in private dental practice were satisfied with the prosthesis, but a substantial amount of dissatisfaction existed. Dissatisfaction was related to age, health, prior experience with a prosthesis, and the type of opposing dentition.	United States
Celebić and Knezović-Zlatarić (2003) [28]	Retrospective study	268	63 (67 CD)	35 men (RPD), 57 men (CD), 99 women (CD)	RPD (retained clasps)	CD (156)	-	Analog scale	Satisfaction	CD wearers were significantly more satisfied than RPD wearers with speech, chewing and retention of maxillary denture, while RPD wearers were significantly more satisfied with the retention and the comfort of wearing mandibular denture (*P *< 0.05).	Croatia
Zlatarić and Celebić (2008) [29]	Multiple regression analysis	103	63	35 men	RPD		Kennedy Class I	Analogue scale	General patient satisfaction and patient satisfaction with aesthetics, retention, speech, chewing, and comfort	Aesthetics, chewing, and speech had significant effects on the patient’s general satisfaction with dentures.	Croatia
Jensen et al. (2016) [30]	Retrospective study	21	-	-	ISRPD		Kennedy class I	VAS	Satisfaction outcomes	The patients scored 8.4 (SD 2.1) on general satisfaction on a 0–10 numeric scale meaning highly satisfied	The Netherlands
Waas et al. (1994) [31]	Cross-sectional study	320	55-75	-	RPD (acrylic resin [>50%], distal extension [60%])	-	-	Questionnaire	Satisfaction with the functioning of the dentition, chewing, aesthetics, speech and comfort	No differences in satisfaction were found between subjects with a metal frame prosthesis and an acrylic denture, nor between tooth-bounded and distal extension prostheses. The conclusion can be drawn that the indication for an RPD. should be limited in elderly people unless the RPD increases the number of the occlusal units	The Netherlands
Frank et al. (2000) [3]	Retrospective study	82	-	-	RPD	-	-	Questionnaire	Clinical acceptability and satisfaction	The standards appear to be unrelated to patient satisfaction. The most important standards are rest form, base extension, and stress distribution.	United States
Threeburuth et al. (2008) [32]	Randomized clinical trial	30	64.1 ± 5.56	10 men	RPD (immediate-load mini implants, conventional-size implants)	-	Kennedy Class I	VAS	Comfort, retention, and chewing performance	Overall patient satisfaction with implant retained RPDs was significantly higher than with conventional RPDs in both groups (*P* < .05). Immediate-load mini dental implants can be used to retain mandibular Kennedy class I RPDs successfully if high primary stability is achieved.	Thailand
Alqutaibi (2020) [33]	Randomized crossover trial	10	58.7 ± 9.3	3 men	RPD (conventional for three months followed by attachment retained for three months)	-	-	VAS	Satisfaction outcomes; cleaning, comfort, aesthetics, stability, mastication, oral condition, and general satisfaction	Higher short-term satisfaction rates in patients with ARRPDs than with the conventional clasp-retained RPDs. The superior aesthetics of ARRPDs are recognized.	Saudi Arabia
Kapur et al. (1991) [34]	Retrospective study	228	-	-	RPD (115)	FPD (113)	Kennedy Classes I and II	Satisfaction Questionnaire	Satisfaction, chewing, comfort, and safety	Both groups perceived improvement in chewing ability, comfort, and ease of chewing, eating enjoyment, and feeling secure with prostheses. Results support the superiority of the FPD in terms of patient satisfaction, but not enough to favor this type of prosthesis over the RPD without consideration of other pertinent factors.	United States
Zlatarić et al. (2003) [16]	Retrospective study	205	38-89	80 men	RPD (123 maxillary, 138 mandibular)	-	Kennedy Class I (57.4%) and Class II (28.8%)	Satisfaction Questionnaire	Patient satisfaction	Majority of the patients treated with RPDs were satisfied with the prosthesis. Dissatisfaction was related to mastication, aesthetics, number of missing teeth, and maintenance of oral hygiene.	Croatia
Wakabayashi et al. (1998) [35]	Retrospective study	66	61.2	24 men	RPD (50 maxillary, 44 mandibular)	-	-	VAS	aesthetics, pain, comfort, stability, ability to speak, chew, and general satisfaction	Female rated lower satisfaction with the comfort of their dentures, Younger patients expressed less satisfaction with the aesthetics than the older patients. The period of wearing dentures correlated with psychological measures of pain, comfort, and general satisfaction. No correlation between general satisfaction with dentures and retention.	Japan
Cosme et al. (2006) [36]	Retrospective study	50	36-76	11 men	RPD	-	Kennedy Class I [20]	Satisfaction Questionnaire	Patient satisfaction	More than 50% of patients classified their RPDs as excellent regarding retention, mastication, aesthetics, comfort, and hygiene. In the professional evaluation, retention and stability were considered excellent in more than 66% of cases, and hygiene of teeth and prostheses was considered good in 52% and 46%, respectively.	Brazil
Al Omiri et al. (2013) [37]	Retrospective study	68	53.2 ± 11.8	38 men	RPD [36]	CD [32]	-	Dental Impact on Daily Living Questionnaire	Patient satisfaction	Patients’ satisfaction with oral condition improved following using removable prosthetic rehabilitation with RPD having better impacts than CD. Psychological profiles might play a role and explain prosthetic impacts on daily living and patients’ satisfaction with prostheses	Saudi Arabia
Alageel et al. (2019) [38]	Retrospective study	75	-	-	RPD (retention)	-	-	McGill Denture Satisfaction Instrument	Patient satisfaction (retention)	RPD retention predicted from the number and position of clasps and missing teeth might help to determine patient satisfaction. In addition, patient satisfaction with RPDs was influenced by the arch type, the presence of a distal extension base, and the number of clasps	Canada
Hundan and Madan (2012) [39]	Retrospective study	30	25-45	-	RPD (flexible and conventional)	-	Kennedy Class II	Satisfaction Questionnaire	Aesthetics, and oral soft tissue tolerance	Statistically significant results were obtained in favor of flexible RPDs, in the parameters of ‘aesthetics’ and ‘overall patient satisfaction’.	India
Hartog et al. (2014) [40]	Prospective study	153	38.3 ± 14.7	45.7% men	RPD (implant-supported, acrylic resin tissue-supported)	-	-	VAS, Satisfaction Questionnaire	Satisfaction (comfort, function, and aesthetics)	Patient satisfaction with a single-tooth implant in the aesthetic zone is high. Compared with an RPD that patients wore before implant treatment, patient satisfaction improved significantly after implant treatment in terms of function, comfort, and aesthetics	The Netherlands
Zlatarić et al. (2000) [41]	Retrospective study	165	38-87	59 men	RPD	-	-	Satisfaction Questionnaire	Comfort, function, aesthetics, and general satisfaction	Women were more satisfied with chewing with lower partial dentures than men. Patients with more missing teeth gave lower grades for the comfort of wearing dentures. Patients of higher education gave lower grades for aesthetics. Dissatisfaction was related to mastication, aesthetics, the number of missing teeth, and the ability to speak.	Croatia
Khan et al. (2017) [42]	Cross-sectional study	80	57.4 ± 13.1	-	RPD	-	-	Satisfaction Questionnaire	Patient satisfaction (eating, smiling, emotional, contact with family)	Post-treatment, 76.3% indicated good oral health and satisfaction with no signiﬁcant differences between the three Kennedy groups.	South Africa
Hakestam et al. (1997) [43]	Clinical follow-up study	42	-	-	RPD	FPD	-	Satisfaction Questionnaire, California Dental Association (CDA) quality assessment system	Patient satisfaction	The RPDs had a somewhat higher share of nonacceptable appliances according to the CDA criteria. There was an association between the CDA categories and patient satisfaction.	Sweden
Almohsen and Mahmoud (2021) [44]	Randomized cross-sectional study	60	52.25 ± 1.8	60 men	RPD	-	-	Satisfaction Questionnaire	Satisfaction in chewing, speech, appearance, taste, pain, digestion, and psychological measurements	Most patients were satisfied with their properly fabricated RPDs in almost all seven categories. No significant differences in satisfaction were found regarding the type of the arch, age, and time after delivering the denture	Saudi Arabia
Sadek and Elawady (2019) [45]	Randomized Controlled Trial	42	-	-	RPD (Thermo press, conventional Vitallium)	-	Kennedy Class II	VAS	Satisfaction	Patient satisfaction and abutment survival were better with Thermo press RPD than conventional Vitallium RPD or Vitallium RPD with a surveyed bridge restoring the modification area. Although a nonstatistically significant difference was found in the survival rate of abutments between groups, a clinically important result was revealed as no abutment failures were reported in the Thermo press group.	Saudi Arabia
Persic et al. (2015) [46]	Cross-sectional study	150	61-84	72 men	Maxillary RPD (c_RPD (88), PA-RPD (62))	-	-	Orofacial Esthetic Scale, CFQ	Aesthetics, chewing function	Treatment outcomes were better in the PA-RPD group than the CRPDs. Women showed greater concern for the treatment outcomes; their rates were significantly better than in male patients in the PA-RPD group; however, when their satisfaction was lower, their rates were significantly worse than in male patients (in the C-RPD group).	Switzerland
Bortolini et al. (2010) [47]	Cross-sectional study	32	-	-	RPD (implant-retained)	-	-	Satisfaction Questionnaire	Patient satisfaction	Implant-retained RPDs are a reliable intermediate solution that can reduce biological and economic costs while maintaining implant treatment benefits and the ease of RPD procedures.	Italy
Wismeijer et al. (2011) [48]	Prospective study	48	-	-	RPD (Implant-assisted mandibular bilateral distal extension)	-	-	VAS	Patient satisfaction	There were significantly improved parameters of overall satisfaction, stability, chewing and appearance after three years (*P* < 0.05). There were also improvements in stability, chewing, and overall satisfaction. Speech also improved, but not significantly. Ball abutments (retentive anchors) on the distal implants, as opposed to healing caps, improved patient satisfaction for stability, chewing, and overall satisfaction.	New Zealand
Wolfart et al. (2016) [49]	Prospective clinical study	30	64 ± 6	8 men, 9 female (RPDP)	RPD	-	-	VAS	Chewing satisfaction	All patients, were very satisfied after therapy concerning the ability to speak, chew, and stability of their prosthesis. Patients with a strongly reduced dentition and edentulous patients benefit from strategically placed implants under the existing removable dentures.	Germany
Manzon et al. (2019) [50]	Retrospective study	120	73	-	VALPLAST-RPD (Polyamide VALPLAST), CoCr-RPD (cobalt chromium alloy), and PMMA-RPD (heat polymerized)	-	-	Satisfaction Questionnaire	Patient satisfaction, including aesthetic, functional, and clinical outcomes	VALPLAST-RPD was the most satisfactory aesthetically. Patients with PMMA-RPD claimed a higher level of encumbrance (*P *< 0.001) and increased speech difficulties (*P *= 0.002). Each RPD material utilized may present advantages and disadvantages in an elderly population.	Italy

Study characteristics: a summary

Five of the included studies were randomized clinical studies [20,32-33,44-45]. Four of the included studies were prospective clinical studies [24,40,49], and one included study was a population-based study [27]. The rest of the included articles were retrospective studies or clinical follow-up studies. Only 10 of the included studies had a sample population of more than 100 patients, with a total of 1,844 patients [16,18,28-29,31,34,40-41,46,50]. Two of the included studies compared RPDs with complete dentures (CDs) [28,37], and two studies compared RPDs with fixed partial dentures (FPDs) [34,43].

General satisfaction

The results from various studies examining satisfaction with RPDs reveal a high rate of satisfaction among patients. Studies reported an overall above-average rate of satisfaction among patients. Aljabri et al. reported 80% satisfaction [19], Shala et al. reported 73.6% satisfaction among first-timers [21], Waas et al. reported 81% satisfaction [31], Zlataric et al. reported 60% satisfaction [41], and Cosme et al. reported more than 50% being satisfied [36]. Other studies similarly reported high levels of general satisfaction among patients wearing RPDs [3,16,27,37,38].

The satisfaction scales that were used vary among articles. All the articles used questionnaires that were completed by the patients in different stages of the treatment. Some articles designed their questionnaires, and other articles used established tools that were already available to assess the satisfaction rates. The most used satisfaction scale was the visual analog scale (VAS), which is a famous scale consisting of a 10 cm horizontal line with two descriptors at the beginning and the end representing the maximum and minimum satisfaction rates. Also scales like the dental impact on daily life (DIDL), McGill Denture Satisfaction Instrument, oral health-related quality of life (OHRQoL) measures, and Chewing Function Quality (CFQ), the Orofacial Esthetic Scale (OES-CRO) were used.

Factors influencing RPD satisfaction

Gender

Frank et al. and Zlataric et al. found no significant difference in the level of satisfaction between men and women [19,27,16,41]. Yoshimoto et al. studied masticatory satisfaction among patients wearing RPD [18]. The authors established that masticatory satisfaction was not associated with gender. Akinyamoju et al. found a gender difference in the satisfaction of the subjects regarding the appearance of the dentures [22]. Females gave a higher mean rank score in terms of satisfaction with the appearance of acrylic partial dentures compared to men. This was also the case for flexible partial dentures. Al Omiri et al. found that females were less satisfied with appearance [37]. Akinyamoju et al. reported that women exhibited a higher mean satisfaction for overall comfort with eating in comparison to men [22]. Wakabayashi et al. reported significantly lower satisfaction among women regarding comfort [35].

Zlataric et al. reported that men were less satisfied with mandibular RPD in terms of mastication as compared to women [16,41]. Persic et al. found that gender alone did not have a significant impact. However, when combined with the factor of RPD retention type, it yielded significant effects. Female patients reacted more intensely than male patients for both RPD groups [46]. Khan et al. reported that, despite women being the majority, most complaints regarding different impacts came from men [42].

Age

Frank et al. reported that older adults were more satisfied with their RPDs except for subjects with opposing RPDs [27]. The authors reported that people younger than 60 expressed more dissatisfaction compared to those above 60. Waas et al. reported that older subjects with an RPD were in general less satisfied and reported more problems than subjects without RPDs [31]. Aljabri et al. found no significant difference in satisfaction rates between patients aged above and below 50 years [29]. Almohsen and Mahmoud found no significant difference in all criteria between patients above 50 and below 50 in all criteria except regarding digestion and taste where older patients were more satisfied [44]. Al Omiri et al. found no significant relationship between age and satisfaction [37].

Akinyamoju et al. found that subjects aged 36-45 years (36.7%) were more satisfied with the appearance of acrylic dentures [22]. Koyama et al. established a statistically significant correlation between age and satisfaction [26]. Wakayabashi et al. found that younger patients were less satisfied with the aesthetics of their dentures compared with older adults [35].

Social Classes

Akinyamoju et al. found that subjects in the lower class were more satisfied with the appearance of the acrylic denture (*P *= 0.61). Other studies have reported an insignificant influence of socioeconomic status on RPD satisfaction [22].

Experience With RPD

Frank et al. reported that patients who had experience with previous RPDs were more satisfied than the patients with their first-ever RPDs [3,27]. Zlataric et al. found no difference in satisfaction between patients with their first-ever RPDs and those who had previous experience [16,41]. Wakayabashi et al. established that the more experience patients had with their RPDs, the more they were satisfied with their ability to speak [35]. They also reported that patients who visited the clinic shortly after undergoing RPD therapy reported more problems with comfort and pain and were unhappy compared to long-term users. Almohsen and Mahmoud found no significant difference in time of use (less or more than 12 months) and level of satisfaction except in the psychological aspect [44].

Missing Teeth and Classification

Zlatarić et al. reported that patients with many missing teeth in the lower jaw (group 3, more than 10 missing) were less satisfied with comfort in comparison with patients with fewer missing teeth [41]. Zlatarić et al. established that patients with a greater number of missing teeth in the mandible had more uncomfortable RPDs in comparison with the patients with fewer missing teeth [16]. Alageel et al. found that a larger proportion of patients were satisfied with maxillary RPDs compared to mandibular RPDs. Additionally, patients with five missing teeth were more satisfied compared to those with six or more missing teeth [38].

Wakayabashi et al. reported that Kennedy Class IV patients had lower satisfaction with aesthetics compared to Kennedy Classes I and II patients. They also found that patients expressed dissatisfaction with aesthetics in dentures that involved anterior teeth [35]. Almohsen and Mahmoud reported that Kennedy Class III patients had a higher satisfaction rate regarding speech. Kennedy Class III and those with modifications had higher satisfaction regarding digestion and taste [44].

Types of RPD

Aljabri et al. and Akinyamoju et al. found a significant difference in patient satisfaction for metal and flexible RPDs [19,22]. This was about appearance, speech, and comfort while eating. Akinyamoju et al. reported that more subjects were satisfied with flexible dentures in terms of aesthetics, speech, and comfort [22]. Hundan and Madan found flexible dentures superior over casted ones regarding overall satisfaction [39]. Fueki et al. found that thermoplastic resin-removable partial dentures (TR-RPDs) had higher overall satisfaction scores compared to conventional metal clasp-retained removable partial dentures (MC-RPDs) [20]. Satisfaction was a result of appearance, comfort, speech, food impaction, ease of cleaning, denture stability, and mucosal pain. Alqutaibi reported a higher satisfaction rate with ARRPDs compared with conventional RPDs [33]. This was regarding ease of cleaning, speech, comfort, aesthetics, masticatory ability, and stability. Sadek et al. found thermo press RPD with more overall satisfaction compared to conventional RPDs [45]. It had increased retention and adaptation to underlying tissues. Manzon et al. determined that VALPLAST RPD was the most satisfactory for the elderly in terms of aesthetics [50]. PMMA RPD (polyamide) was, however, more suitable for this population considering their decreased masticatory force and softer diets compared to younger populations.

Attachments

Shala et al. reported higher levels of satisfaction for RPDs with attachment compared with RPD with clasps [21]. Alageel et al. reported that patients were more satisfied with supported RPDs than those with distal extension bases [38]. This was in terms of oral condition, mastication, appearance, and retention. Also, RPDs with more than three clasps had higher satisfaction compared with those with two or fewer. Patients were highly satisfied with chewing ability, retention, and aesthetics. Negoro et al. reported on implant-assisted removable partial dentures (IARPDs) with a magnetic attachment, which scored significantly better overall patient satisfaction compared to IARPD with a healing cap [24].

Hartog et al. reported significant improvement in satisfaction with aesthetics, function, and comfort for patients who received implant treatment [40]. Bortolini et al. reported increased patient satisfaction with the combined use of implants and conventional RPDs [47]. Wismeijer et al. studied mandibular implant-assisted RPD and reported improved overall satisfaction, chewing, and stability [48]. Wolfart et al. established that strategic placement of implants resulted in improved masticatory satisfaction when placed under RPD [49].

Complaints

Aljabri et al. reported complaints from 26.7% of the participants regarding pain during eating and speech problems [19]. Koyama et al. found a statistically significant correlation between patient satisfaction and pain, color of denture, and tooth shape [26]. Frank et al. reported that dissatisfaction mainly stemmed from chewing, mouth cleanliness, speech, appearance, and RPD cleanliness [27]. Wakabayashi et al. reported less complaints from patients regarding pain [35]. 

Comparison

Celebić and Knezović-Zlatarić reported that CD wearers were significantly more satisfied than RPD wearers in terms of chewing, speech, and retention [28]. RPD wearers were more satisfied in terms of retention and comfort. There was no difference regarding general satisfaction. Al Omiri et al. reported greater satisfaction for patients with partial dentures compared to CDs [37]. Kapur et al. found FPDs superior in terms of patient satisfaction; however, RPD was superior when other pertinent factors were considered [34]. Hakestam et al. used a quality assessment scale and found that high-quality assessment scores were associated with more satisfaction; RPDs compared to FPDs had less desirable quality factors [43].

Summary of results

This systematic review examined overall satisfaction with RPDs and explored how factors such as gender, age, social class, experience with RPDs, number and classification of missing teeth, types of RPDs, and attachments influenced patient satisfaction. The overall results suggested high satisfaction rates [3,19,21,31,36-38,41-42]. The reported satisfaction rates were 80% [19,21], 81% [31], 60% [41], and >50% [36]. Age was found to impact satisfaction, with older adults generally expressing higher levels of satisfaction. Gender differences were also observed, particularly in the context of appearance satisfaction [22,35]. Prior RPD experience correlated positively with satisfaction [15,27,41], and the number and location of missing teeth influenced satisfaction [15,38,41]. The type of RPD significantly affected satisfaction, with notable differences between metal and flexible RPDs [19-20,22]. Attachments, such as magnetic attachments and implants, increased satisfaction [21,40]. Patient complaints varied, with pain, aesthetics, and cleanliness being common sources of dissatisfaction [19,26,41]. These findings show the need for a personalized approach in RPD treatment to optimize patient satisfaction and OHRQoL.

Discussion

RPDs are a prevalent and adaptable prosthodontics treatment modality, addressing the problems of partial edentulism and resulting in the restoration of oral function and aesthetics among different populations. Prostheses are commonly used by patients with several missing teeth but retain some natural dentition. They are designed to enhance mastication, preserve the remaining teeth, and restore the integrity of the oral cavity. The success of the use of RPDs extends beyond the technicalities of their design and fabrication to include aspects of **oral health quality of life** and patient satisfaction. The use of RPDs should feel as natural as possible for patients, and examining the narratives of patients who wear them is critical in understanding areas for improvement. Patient satisfaction is a complex construct that assesses individual subjective experiences, perceptions, and contentment with oral health-related outcomes. Several factors contribute to patient satisfaction with RPDs, including fit and comfort, aesthetics and appearance, functional performance, psychosocial effect, and prior experience and expectations.

This study established that the majority of the patients are satisfied with their RPDs [3,16,19,21,27,31,36-38,41-42], with most reviewed studies reporting an above-average general satisfaction. Several factors contribute to the lack of patient satisfaction in the use of RPDs, and all this relates to the age of the patient, retention of RPD, poor denture fit, and adaptive capacity. Ill-fitting dentures can lead to discomfort, pain, and compromised functionality. Dentures that enhance, rather than hinder, mastication and speech functions contribute to an overall positive experience among patients. Psychosocial issues such as self-esteem, confidence, and the ability to engage socially are influenced by the denture's impact on oral function and aesthetics. Patients' prior experiences with dental prosthetics and their expectations regarding RPD outcomes play a role in shaping satisfaction. Aligning patient expectations with realistic outcomes is pivotal for achieving high levels of satisfaction.

Age emerged as a noteworthy factor influencing RPD satisfaction. Frank et al. noted that older adults generally expressed higher satisfaction, with dissatisfaction more prevalent among individuals under 60, especially in cases of opposing RPDs [27]. However, Waas et al. reported that older subjects were less satisfied [31]. Other studies, such as those by Almohsen and Mahmoud and Akinyamoju et al., found no consistent correlation between age and satisfaction [44,22]. The nuanced relationship between age and satisfaction underscores the need for a personalized approach to address the diverse preferences of different age groups.

The influence of gender on RPD satisfaction appeared to vary across different aspects. Studies by Frank et al., Aljabri et al., and Zlatarić et al. found no substantial difference in overall satisfaction between men and women [27,19,41,16]. However, disparities arise when specific elements are considered. Akinyamoju et al. observed that women exhibited higher satisfaction with the appearance of both acrylic and flexible partial dentures [22]. Conversely, Wakabayashi et al. reported lower satisfaction among women regarding comfort [35]. These findings suggest that gender-related factors can play a role in specific facets of RPD satisfaction.

Experience with RPDs emerged as a consistent predictor of satisfaction. Patients with prior RPD experience, as reported by Frank et al. and Zlatarić et al., tended to express higher satisfaction than those with their first-ever RPDs [27,16,41]. Long-term users, as highlighted by Wakayabashi et al., demonstrated increased satisfaction, particularly in terms of speech [35]. The positive correlation between experience and satisfaction emphasizes the importance of patient adaptation and acclimatization over time.

The number and location of missing teeth seemed to play a crucial role in RPD satisfaction. Zlatarić et al. observed that patients with more missing teeth, especially in the mandible, reported lower satisfaction, highlighting the importance of denture stability and comfort [16,41]. The classification of RPDs, such as Kennedy Class, also influences satisfaction, with Kennedy Class IV patients expressing lower satisfaction with aesthetics, as noted by Wakayabashi et al. [35].

The type of RPD significantly affected satisfaction rates. Studies by Aljabri et al. and Akinyamoju et al. revealed notable differences in satisfaction between metal and flexible RPDs, particularly in appearance, speech, and comfort while eating [19,22]. Fueki et al. reported higher overall satisfaction with thermoplastic resin RPDs compared to metal-cast RPDs, citing factors such as appearance, comfort, and stability [20].

The use of attachments, such as magnetic attachments and implants, significantly impacted satisfaction. Shala et al. found higher satisfaction for RPDs with attachments compared to those with clasps [21]. Hartog et al. reported improved satisfaction with aesthetics, function, and comfort for patients who received implant treatment, suggesting a positive correlation between implant-assisted RPDs and overall satisfaction [40].

The satisfaction rates of patients using RPDs are shown to be influenced by several factors. These findings highlight the importance of individualized approaches in RPD treatment, considering the diverse preferences and needs of patients across different demographic and clinical contexts. Further research is needed to better understand the complexities of these factors and how they affect RPD satisfaction. 

The heterogeneity in the scales used to evaluate patient satisfaction was observed as a limitation, hindering meta-analysis. Further studies are needed to standardize the tools assessing patient satisfaction and correlate them with other factors. Additionally, further work is needed to evaluate the interactions between the different variables affecting patient satisfaction. This would enable a better understanding of the most important factors in satisfaction and their interactions.

## Conclusions

In conclusion, this systematic review provides valuable insights into the satisfaction of patients with RPDs and the factors influencing their experiences. The overall findings indicate a high level of satisfaction among RPD wearers, with reported satisfaction rates ranging from 50% to 81% across various studies. The study characteristics, including the diverse methodologies employed, contribute to a comprehensive understanding of the factors influencing satisfaction.

Age emerged as a notable factor affecting RPD satisfaction, with older adults generally expressing higher levels of satisfaction, particularly when not dealing with opposing RPDs. Gender differences played a role in specific aspects of satisfaction, such as appearance, with women tending to exhibit higher satisfaction in this regard. Experience with RPDs consistently correlated with higher satisfaction, emphasizing the importance of patient adaptation and acclimatization over time.

The number and location of missing teeth, as well as the classification of RPDs, demonstrated significant impacts on satisfaction. Patients with more missing teeth, especially in the mandible, reported lower satisfaction, highlighting the importance of denture stability and comfort. The type of RPD, whether metal or flexible, significantly influenced satisfaction rates, with thermoplastic resin RPDs often being associated with higher overall satisfaction.

The use of attachments, such as magnetic attachments and implants, emerged as a critical factor positively impacting satisfaction. RPDs with attachments, as well as implant-assisted RPDs, were associated with higher levels of overall satisfaction, suggesting the potential benefits of incorporating advanced prosthodontic techniques.

Complaints from patients, including issues related to pain, aesthetics, and cleanliness, underscore the need for continued improvement in RPD design and fabrication. These findings collectively emphasize the importance of individualized approaches in RPD treatment, considering the diverse preferences and needs of patients across different demographic and clinical contexts.

In conclusion, while the overall satisfaction with RPDs is encouraging, further research is warranted to delve deeper into the complexities of these factors and their interplay to optimize patient satisfaction and OHRQoL in RPD wearers.
